# Normothermic Regional Perfusion and Hypothermic Oxygenated Machine Perfusion for Livers Donated After Controlled Circulatory Death With Prolonged Warm Ischemia Time: A Matched Comparison With Livers From Brain-Dead Donors

**DOI:** 10.3389/ti.2022.10390

**Published:** 2022-04-22

**Authors:** Damiano Patrono, Marinella Zanierato, Marco Vergano, Chiara Magaton, Enrico Diale, Giorgia Rizza, Silvia Catalano, Stefano Mirabella, Donatella Cocchis, Raffaele Potenza, Sergio Livigni, Roberto Balagna, Renato Romagnoli

**Affiliations:** ^1^ General Surgery 2U–Liver Transplant Unit, A.O.U. Città della Salute e della Scienza di Torino, University of Turin, Turin, Italy; ^2^ Department of Anesthesia and Critical Care, Azienda Ospedaliera Universitaria Città della Salute e della Scienza di Torino, Turin, Italy; ^3^ Department of Anesthesia and Intensive Care, San Giovanni Bosco Hospital, Turin, Italy; ^4^ Regional Procurement Organization, Azienda Ospedaliera Universitaria Città della Salute e della Scienza di Torino, Turin, Italy; ^5^ Anesthesia Department 2, A.O.U. Città Della Salute e Della Scienza di Torino, Turin, Italy

**Keywords:** donation after circulatory death, abdominal normothermic regional perfusion, hypothermic oxygenated machine perfusion, warm ischemia time, ischemic cholangiopathy, liver transplantation outcome

## Abstract

Prolonged warm ischemia time (WIT) has a negative prognostic value in liver transplantation (LT) using grafts procured after circulatory death (DCD). To assess the value of abdominal normothermic regional perfusion (A-NRP) associated with dual hypothermic oxygenated machine perfusion (D-HOPE) in controlled DCD LT, prospectively collected data on LTs performed between January 2016 and July 2021 were analyzed. Outcome of controlled DCD LTs performed using A-NRP + D-HOPE (*n* = 20) were compared to those performed with grafts procured after brain death (DBD) (*n* = 40), selected using propensity-score matching. DCD utilization rate was 59.5%. In the DCD group, median functional WIT, A-NRP and D-HOPE time was 43, 246, and 205 min, respectively. Early outcomes of DCD grafts recipients were comparable to those of matched DBD LTs. In DCD and DBD group, incidence of anastomotic biliary complications and ischemic cholangiopathy was 15% versus 22% (*p* = 0.73) and 5% versus 2% (*p* = 1), respectively. One-year patient and graft survival was 100% versus 95% (*p* = 0.18) and 90% versus 95% (*p* = 0.82). In conclusion, the association of A-NRP + D-HOPE in DCD LT with prolonged WIT allows achieving comparable outcomes to DBD LT.

## Graphical Abstract

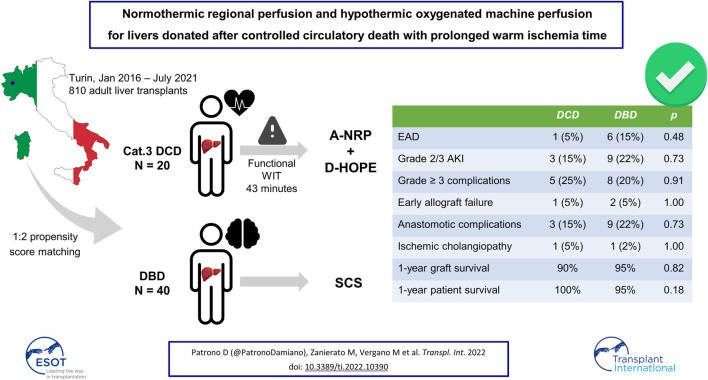

## Introduction

In liver transplantation (LT) using grafts from donors whose death has been determined by circulatory criteria (DCD), warm ischemia time (WIT) has a major impact on the outcome. Prolonged WIT has consistently been associated with an increased risk of primary non-function, ischemic cholangiopathy (IC) and inferior graft survival (1–5). In contrast with most countries with active DCD transplant programs, Italian law requires a 20-min period of absent cardiac electrical activity for death declaration (6), which significantly increases the risks associated with the use of these grafts and has slowed down implementation of DCD LT in Italy (7).

However, mainly prompted by the favourable Spanish experience with the use of abdominal normothermic regional perfusion (A-NRP) to recover DCD liver grafts from Maastricht category 2 donors (8), DCD LT was introduced in Italy in 2015 (9, 10). Given the unique characteristics of the Italian setting, use of A-NRP has been established as mandatory, while subsequent ex-situ machine perfusion (MP) has been encouraged and adopted by most centres.

A growing body of literature supports the benefits of A-NRP for livers procured from Maastricht category 3 (controlled) DCD donors (11–17). In the same setting, use of end-ischemic (dual)-hypothermic oxygenated machine perfusion (HOPE/D-HOPE) has been consistently associated with better liver graft function and lower incidence of IC as compared to static cold storage (SCS) (18–21). However, these studies reported shorter WIT compared to what can possibly be achieved in Italy.

In the Italian setting, previous studies have shown that the association of A-NRP with ex-situ machine perfusion for controlled DCD liver grafts allows achieving good LT outcomes (22–24), which appear to be comparable to those of DCD livers preserved by ultra-rapid recovery and preserved by SCS (25). However, a formal comparison with LT using livers from donors after neurologic determination of death (DBD) accounting for potential confounders and demonstrating comparable outcomes is still lacking.

Thus, the aim of the study was to report our results with the use of A-NRP + D-HOPE for controlled DCD liver grafts with prolonged WIT. To assess the effectiveness of this approach, outcomes of DCD grafts recipients were compared to those of a matched cohort of DBD LTs, selected using propensity score matching.

## Materials and Methods

Prospectively collected data on adult (≥18-year-old) patients who underwent LT at our centre from January 2016 to July 2021 were retrospectively analyzed. Collected data included donor and recipient baseline characteristics, operational details, and prognostic scores (26, 27). The UK-DCD risk score (4), a prognostic score for DCD LT based on 4 donor and 3 recipient variables, was used to grade the risk profile associated with each case. Recipients of a combined transplant, retransplant, partial graft or suffering from on-table death were excluded. To limit confounding, recipients of a DBD graft treated with any type of machine perfusion were also excluded, as well as recipients of Maastricht category 2 DCD grafts and of Maastricht category 3 DCD grafts treated with a machine perfusion modality other than D-HOPE. To control selection bias, two comparable cohorts of DBD and controlled DCD LTs were selected using 1:2 propensity score matching. Minimal patient follow-up was 6 months. The study was conducted according to the principles of the Istanbul and Helsinki declarations and was approved by the ethics committee of our Institution (protocol 506/2021).

**FIGURE 1:**
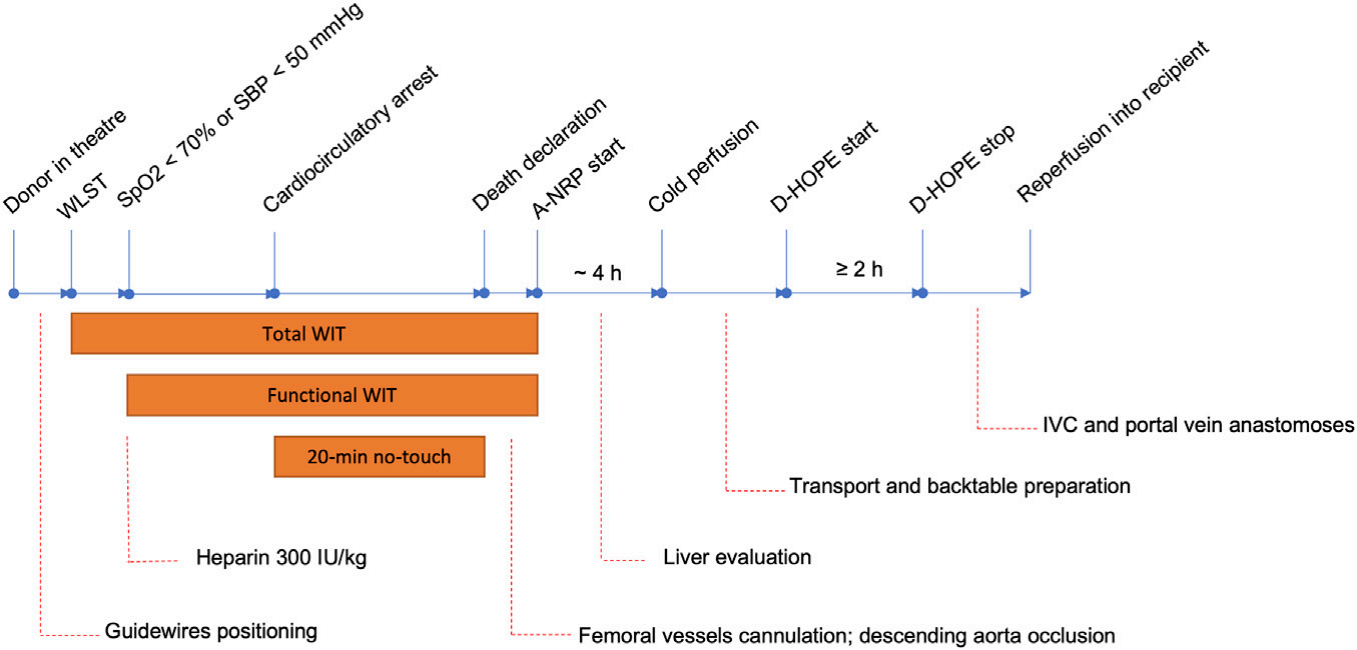
DCD procurement protocol. Abbreviations: WLST, withdrawal of life-sustaining treatment; SBP, systemic blood pressure; A-NRP, abdominal normothermic regional perfusion; D-HOPE, dual hypothermic oxygenated machine perfusion; WIT, warm ischemia time; IVC, inferior vena cava.

Our procurement and machine perfusion protocols are depicted in Figure 1. Briefly, withdrawal of life-sustaining treatment (WLST) took place in the operating theatre, after guidewires for subsequent femoral vessels cannulation had been placed under ultrasound guidance (pre-mortem cannulation is not allowed in Italy). At the onset of functional warm ischemia (peripheral O_2_ saturation ≤70% or systolic blood pressure ≤50 mmHg, whichever occurred first) 300 IU/kg heparin was administered. After 20-min electrical asystole, death was declared, femoral vessels were cannulated and descending aorta was occluded by an endovascular balloon or a surgical clamp, depending on theatre logistic, after which A-NRP was started. During A-NRP, pump flow was maintained ≥1.7 L/min/m^2^ and temperature at 35–36°C (28). Target perfusion pressure was 55–70 mmHg, which was sustained using low dose vasopressin or norepinephrine when necessary, in addition to flow settings and fluid replacement. The circuit sweep gas levels (FiO_2_ and air flow) were adjusted to maintain PaCO_2_ between 35 and 45 mmHg, SaO_2_ about 96–98%, and SvO2 > 60%. If needed, packed red blood cells were transfused to maintain haematocrit ≥20%. Heparin boluses were administered based on activated clotting time values. Blood samples were obtained prior to A-NRP start, at 30 min and then hourly to adjust A-NRP parameters (gas flow, blood flow, FiO_2_, pump speed) and to assess liver injury and function. Target A-NRP duration was 4 h and it was never less than 2 h or more than 6 h. During A-NRP, liver viability assessment was based on a modified version of the criteria proposed by De Carlis et al. (29), including pump flow >1.7 lt/min/m^2^, transaminase level <1,000 IU/L, downward lactate trend, absence of significant (≥15%) macrovesicular steatosis or Ishak >1 fibrosis, good liver and abdominal viscera perfusion, and evidence of bile production. A liver biopsy was systematically obtained to rule out significant necrosis or macrovesicular steatosis. At the end of A-NRP, the liver graft was cold flushed with Celsior (IGL, Lissieu, France) solution through the arterial cannula and trough a portal vein tributary. Liver was prepared on the backtable immediately upon arrival at our transplant centre and subsequently underwent a minimum of 2 h of D-HOPE using the LiverAssist device (XVivo, Groningen, Netherlands), primed with 3 lt of Belzer MP solution (BridgeToLife, Northbrook, IL). Temperature, portal vein and hepatic artery pressure were set at 8–10°C, 3–5 mmHg and 25 mmHg, respectively. D-HOPE was not used with the purpose of viability assessment and all grafts treated by D-HOPE were subsequently transplanted. At the end of recipient hepatectomy, the liver was disconnected from the device and brought to the operating table for implantation.

In DBD group, the liver was flushed with Celsior and preserved by static cold storage until implantation into the recipient. In both groups, the liver was flushed with chilled 5% albumin solution before implantation.

As a rule, liver transplant was performed by the piggyback technique with portal reperfusion first. Following hepatic artery anastomosis, an end-to-end biliary anastomosis was performed using a 2.5 mm T-tube. In all patients graft histology was assessed on time-0 biopsies, which were systematically obtained at the end of transplant operation. Standard immunosuppression included basiliximab, tacrolimus, steroids and mycophenolate mofetil, and was not modified according to treatment group.

Early outcome endpoints included rate of post-reperfusion syndrome (30, 31), transaminase peak, early allograft dysfunction (32), rate and severity of acute kidney injury (AKI) (33), requirement for renal replacement therapy, hospital and intensive care unit (ICU) stay, postoperative complications (34, 35), and the rate of early graft failure (EAF), defined as death of relisting for LT withing 90 days from transplant.

Post-reperfusion syndrome was defined as a drop in mean arterial pression ≥30% from the baseline for at least 1 min and within 5 min from reperfusion (30), whereas severe post-reperfusion syndrome was defined as the onset of severe hemodynamic instability, persistent hypotension, cardiac arrest or hemodynamically significant arrhythmias (31). EAD and AKI were defined according to Olthoff et al. (32) and KDIGO guidelines (33). Postoperative complications were graded according to Clavien-Dindo classification (34), which was also used to calculate comprehensive complication index (CCI) (35).

Biliary complications (36) were diagnosed based on the 3-month cholangiogram obtained before removing the T-tube, or by magnetic resonance cholangiopancreatography (MRCP), which was performed if clinically indicated. Recipients of a DCD graft underwent systematic 6-month and 12-month MRCP.

Variables are presented as number (percentage) of median (interquartile range), as appropriate, and compared using Fisher’s, Chi-square and Mann-Whitney tests. To control selection bias, 1:2 propensity score matching without replacement and using the nearest method was used to select two patient cohorts with comparable characteristics. Variables included in the model were recipient age, body mass index (BMI) and model for end-stage liver disease (MELD) score, hepatocellular carcinoma (HCC) as an indication for LT, donor age and BMI, percentage of macrovesicular steatosis and presence of macrovesicular steatosis ≥15%. Standardized mean differences were used to assess balance obtained by propensity score matching. Patient and graft survival was analyzed using Kaplan-Meier curves. Statistical analysis was performed using R: a language and environment for statistical computing (R Foundation for Statistical Computing, Vienna, Austria).

## Results

During study period, 810 adult LTs were performed, of which 26 using organs proceeding from a DCD donor (cat. 3, *n* = 22; cat. 2, *n* = 4). A total of 37 category 3 DCD donors were signalled in our region during study period, of which 22 were transplanted by our centre. As per Italian regulations, livers from regional DCD donors were allocated locally to our centre, which is the only liver transplant centre in our region, and referred elsewhere only upon refusal by our unit. Four livers were refused based on donor characteristics and the organs were reallocated to other centres. Three of these grafts were successfully transplanted, whereas one was discarded during A-NRP due to elevated transaminases and lack of lactate clearance. Of the remaining 11 livers, 6 were discarded by our and all other Italian centres based on donor features, whereas of 5 offers initially accepted by our centre, 2 were subsequently discarded due to excessive functional WIT, and 3 during A-NRP. The reason to discard the liver during A-NRP was mainly elevated transaminases, which was associated to persistently elevated lactate levels in one case and evidence of gallbladder and bile duct necrosis in another. No liver was discarded based on histological findings. Overall utilization rate of livers from category 3 DCD donors was 25/37 (67.6%), whereas it was 22/37 (59.5%) if we consider only those transplanted at our centre.

**FIGURE 2:**
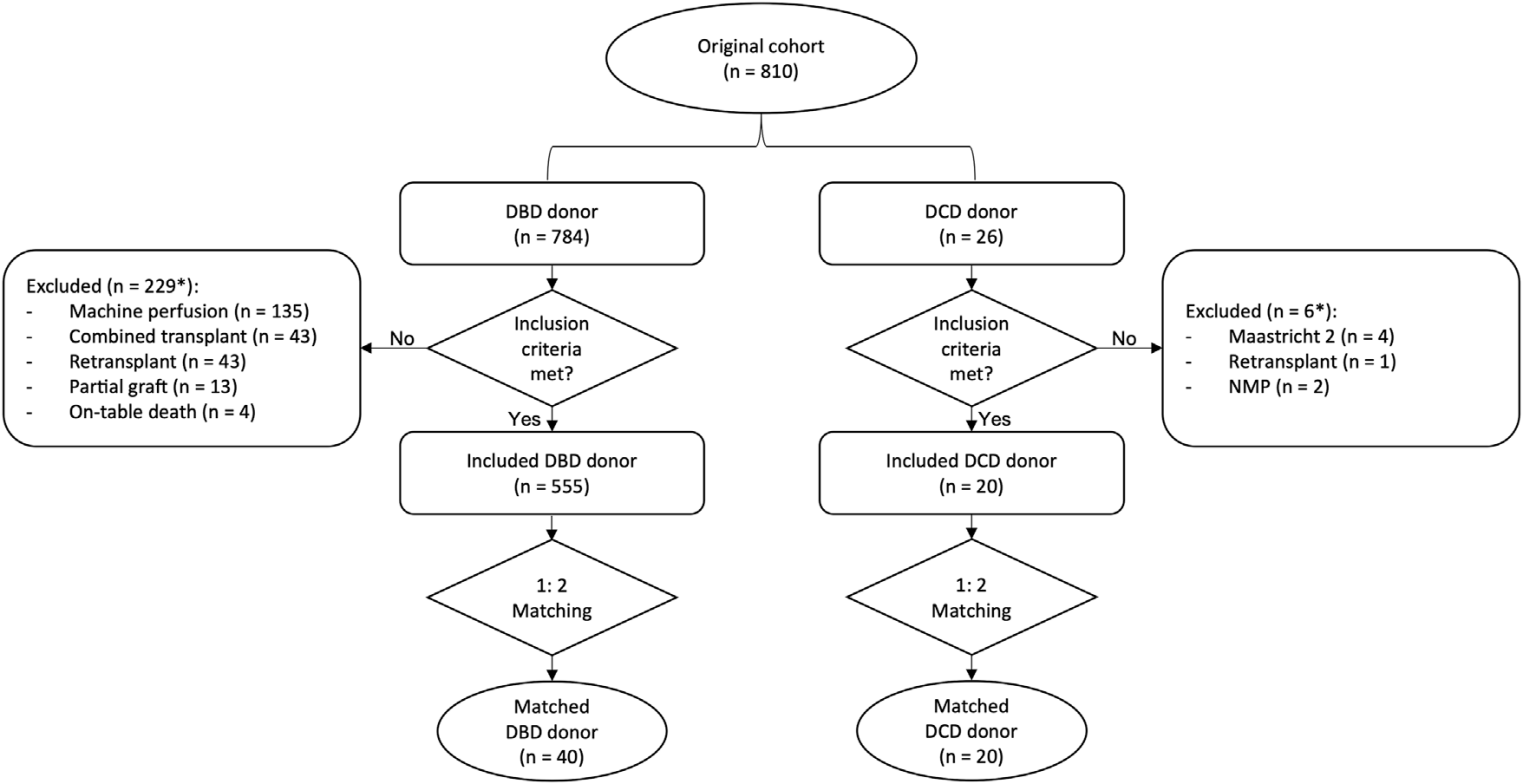
Patient selection flowchart. *some patients met more than one exclusion criterium. NMP, normothermic machine perfusion.

Based on exclusion criteria, 229 and 6 patients were excluded from DBD and DCD group, respectively (Figure 2). In the DCD group, besides 4 recipients of livers from category 2 DCD donors, 2 further cases, including one retransplant, were excluded due to the use of normothermic machine perfusion instead of D-HOPE. Thus, 555 DBD and 20 DCD liver transplants were included for analysis. Finally, outcomes of the 20 DCD LTs were compared to those of 40 recipients of a DBD graft, selected by 1:2 propensity score matching.

**FIGURE 3:**
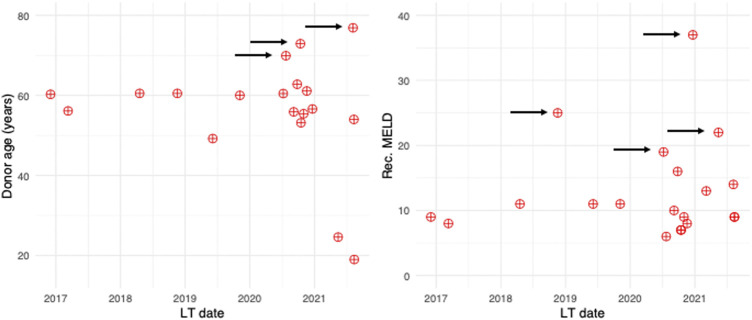
Scatter plot depicting donor age and recipient MELD as a function of study period. During study period, donors of increasing age were considered, and DCD grafts were more frequently allocated to higher-MELD recipients (arrows).

Baseline patient and donor characteristics and operational details are summarized in Table 1. In the DCD group, median donor age and BMI were 60.1 (55.1, 61.5) and 25.0 [23.0, 26.1], and only one liver had 15% macrovesicular steatosis, reflecting our policy of avoiding overlap of additional donor risk factors in this high-risk cohort, characterized by a functional WIT of 43 (35, 46) min. A-NRP and D-HOPE times were 246 (221, 269) and 205 (146, 277) min, respectively. DCD livers were preferentially allocated to low-MELD (10.5 [8.8, 14.5]) patients, with HCC being the indication for LT in 80% of cases. However, with increasing experience, livers from elderly donors were also accepted and procured organs were more frequently allocated to higher-MELD recipients (Figure 3). Despite donor and recipient selection, median UK-DCD risk score (4) was 13 (11, 14) with 17 cases being classified as “futile” and 3 as “high-risk”.

**TABLE 1:** Baseline covariates balance.

	Whole cohort				Matched cohort		
	DBD	DCD	p	SMD	DBD	DCD	SMD
*n*	555	20			40	20	
Rec. age	57.5 [52.4, 62.1]	60.7 [57.4, 66.7]	0.02	0.64	60.6 [56.2, 65.6]	60.7 [57.4, 66.7]	0.04
Gender (male)	404 (73)	16 (80)	0.65	0.17	30 (75)	16 (80)	0.12
Rec. BMI	25.0 [22.7, 27.7]	25.3 [22.6, 27.3]	0.90	0.05	25.2 [22.5, 27.8]	25.3 [22.6, 27.3]	0.01
Indication			0.28	0.65			0.76
Viral hepatitis	276 (50)	9 (45)			27 (68)	9 (45)	
Alcoholic cirrhosis	98 (18)	6 (30)			7 (18)	6 (30)	
Cholestatic disease	39 (7)	2 (10)			0 (0)	2 (10)	
NASH	17 (3)	2 (10)			1 (2)	2 (10)	
Autoimmune	16 (3)	0 (0)			0 (0)	0 (0)	
Acute liver failure	3 (1)	0 (0)			0 (0)	0 (0)	
Other	106 (19)	1 (5)			5 (12)	1 (5)	
MELD	13.0 [9.0, 18.0]	10.5 [8.8, 14.5]	0.17	0.21	11.5 [8.0, 17.2]	10.5 [8.8, 14.5]	0.10
Creatinine (mg/dl)	0.8 [0.7, 1.1]	0.8 [0.7, 1.0]	0.95	0.02	0.9 [0.7, 1.2]	0.8 [0.7, 1.0]	0.20
Dialysis pre-LT	11 (2)	0 (0)	1.00	0.20	1 (2)	0 (0)	0.23
Prev. abdo. surgery	206 (37)	10 (50)	0.35	0.26	21 (52)	10 (50)	0.05
Life support	17 (3)	1 (5)	1.00	0.10	1 (2)	1 (5)	0.13
Ascites	211 (38)	7 (35)	0.96	0.06	14 (35)	7 (35)	<0.01
Encephalopathy	114 (21)	2 (10)	0.38	0.30	7 (18)	2 (10)	0.22
HCC	296 (53)	16 (80)	0.03	0.59	33 (82)	16 (80)	0.06
Donor age	65.4 [52.4, 74.4]	60.1 [55.1, 61.5]	0.13	0.30	63.1 [44.8, 71.7]	60.1 [55.1, 61.5]	0.04
Donor BMI	25.3 [22.9, 27.7]	25.0 [23.0, 26.1]	0.57	0.17	25.3 [23.3, 27.6]	25.0 [23.0, 26.1]	0.14
Macrosteatosis (%)	1.0 [0.0, 5.0]	0.0 [0.0, 1.2]	0.05	0.35	0.0 [0.0, 3.5]	0.0 [0.0, 1.2]	0.02
Macrosteatosis ≥15%	64 (12)	1 (5)	0.57	0.24	2 (5)	1 (5)	<0.01
Microsteatosis (%)	10.0 [1.0, 25.0]	5.0 [0.0, 10.0]	0.04	0.53	10.0 [4.5, 20.0]	5.0 [0.0, 10.0]	0.36
D-MELD	800 [573, 1117]	542 [488, 1014]	0.05	0.33	699 [533, 977]	542 [488, 1014]	0.12
BAR	5.0 [3.0, 19.0]	5.0 [3.0, 8.0]	0.99	0.18	5.0 [3.0, 17.0]	5.0 [3.0, 8.0]	0.09
WIT (min)		43 [40, 48]				43 [40, 48]	
Functional WIT (min)		43 [35, 46]				43 [35, 46]	
A-NRP time (min)		246 [221, 269]				246 [221, 269]	
CIT (min)	431 [379, 482]	261 [229, 295]	<0.01	2.06	418 [375, 510]	261 [229, 295]	1.86
D-HOPE time (min)		205 [146, 277]				205 [146, 277]	
Total pres. time (min)	431 [379, 482]	492 [426, 531]	0.01	0.65	418 [375, 510]	492 [426, 531]	0.58
Portal rep. time (min)	23.0 [21.0, 27.0]	22.0 [20.5, 26.2]	0.47	0.19	23.0 [21.0, 26.2]	22.0 [20.5, 26.2]	0.01
Total rep. time (min)	38.0 [24.0, 50.2]	48.5 [42.0, 59.5]	0.01	0.51	41.0 [24.0, 55.2]	48.5 [42.0, 59.5]	0.41
PRBC units (n)	3.0 [0.0, 8.0]	2.5 [0.0, 7.2]	0.70	0.04	5.0 [0.8, 9.2]	2.5 [0.0, 7.2]	0.01
Graft weight (gr)	1490 [1290, 1720]	1455 [1222, 1610]	0.39	0.19	1475 [1295, 1692]	1455 [1222, 1610]	0.09

Abbreviations: SMD, standardized mean difference; BMI, body mass index; NASH, non-alcoholic steatohepatitis; MELD, model for end-stage liver disease; prev, previous; HCC, hepatocellular carcinoma; D-MELD, donor age * MELD score; BAR, balance of risk score; WIT, warm ischemia time; A-NRP, abdominal normothermic regional perfusion; CIT, cold ischemia time; D-HOPE, dual hypothermic oxygenated machine perfusion; pres, preservation; rep, reperfusion; PRBC, packed red blood cells.

Patient cohorts selected by propensity score matching showed good comparability, as reflected by a standardized mean difference ≤0.10 for all major confounders, including recipient age, BMI and MELD score, HCC as the indication for LT, donor age, graft macrovesicular steatosis, balance of risk (BAR) score and portal reperfusion time (Table 1).

Outcomes in the unmatched and matched cohort are reported in Table 2. Overall, early outcomes in the DCD group were comparable to those observed in the DBD group.

**TABLE 2:** Outcome.

	Whole cohort			Matched cohort		
	DBD	DCD	p	DBD	DCD	p
*n*	555	20		40	20	
Severe PRS	77 (14)	3 (15)	1.00	4 (10)	3 (15)	0.89
End-LT lactate (mmol/l)	2.0 [1.4, 2.8]	1.6 [1.0, 2.4]	0.13	2.0 [1.4, 2.9]	1.6 [1.0, 2.4]	0.26
AST peak (IU/L)	1111 [692, 1752]	761 [589, 1345]	0.13	937 [663, 1438]	761 [589, 1345]	0.63
ALT peak (IU/L)	702 [448, 1126]	461 [385, 608]	0.01	632 [360, 835]	461 [385, 608]	0.18
EAD	157 (28)	1 (5)	0.04	6 (15)	1 (5)	0.48
AKI stage			0.53			0.27
0	178 (32)	8 (40)		10 (25)	8 (40)	
1	226 (41)	9 (45)		21 (52)	9 (45)	
2	107 (19)	3 (15)		4 (10)	3 (15)	
3	44 (8)	0 (0)		5 (12)	0 (0)	
Grade 2/3 AKI	151 (27)	3 (15)	0.34	9 (22)	3 (15)	0.73
Renal replacement therapy	13 (2)	0 (0)	1.00	0 (0)	0 (0)	NA
Early rejection	46 (8)	1 (5)	0.91	3 (8)	1 (5)	1.00
Grade ≥3 complications	126 (23)	5 (25)	1.00	8 (20)	5 (25)	0.91
ICU stay (days)	3.0 [2.0, 5.0]	4.0 [2.0, 5.0]	0.92	4.0 [2.0, 6.0]	4.0 [2.0, 5.0]	0.55
Hospital stay (days)	12.0 [9.0, 17.0]	10.0 [8.0, 19.5]	0.59	12.0 [9.0, 19.0]	10.0 [8.0, 19.5]	0.35
Hospital CCI	22.6 [12.0, 33.7]	16.5 [0.0, 33.9]	0.10	21.8 [8.7, 35.4]	16.5 [0.0, 33.9]	0.26
Early allograft failure	28 (5)	1 (5)	1.00	2 (5)	1 (5)	1.00
Biliary complications						
Anastomotic	85 (15)	3 (15)	1.00	9 (22)	3 (15)	0.73
Fistula	10 (2)	1 (5)	0.85	2 (5)	1 (5)	1.00
Stricture	75 (14)	2 (10)	0.91	7 (18)	2 (10)	0.70
IC	28 (5)	1 (5)	1.00	1 (2)	1 (5)	1.00
Treatment			0.06			0.15
Operational	69 (71)	1 (33)		7 (78)	1 (33)	
Surgery	24 (25)	1 (33)		2 (22)	1 (33)	
Retransplant	4 (4)	1 (33)		0 (0)	1 (33)	
N° of treatments	2.0 [1.0, 3.0]	3.0 [2.5, 4.5]	0.33	2.0 [2.0, 3.0]	3.0 [2.5, 4.5]	0.43
Determining graft loss	5 (1)	1 (5)	0.51	0 (0)	1 (5)	0.72
Determining patient death	1 (0)	0 (0)	1.00	0 (0)	0 (0)	NA

Abbreviations: PRS, post-reperfusion syndrome; LT, liver transplant; EAD, early allograft dysfunction; AKI, acute kidney injury; ICU, intensive care unit; CCI, comprehensive complication index; IC, ischemic cholangiopathy.

In the DCD and DBD group, EAD and grade 2/3 AKI rates were 5% versus 15% and 15% versus 22%, respectively, with no patient requiring renal replacement therapy after LT. Five (25%) and 8 (20%) recipients of a DCD or DBD liver, respectively, developed grade ≥3 complications and median comprehensive complication index was 16.5 (0.0, 33.9) versus 21.8 (8.7, 35.4). Intensive care unit and hospital length of stay was 4 (2, 5) versus 4 (2, 6) and 10 (8, 19.5) versus 12 (9, 19) days, respectively. Two grafts were lost in the DCD group, which were the first and the second in our series. The first graft loss resulted from a hepatic artery injury that occurred during an attempt at performing hepaticojejunostomy for a late biliary fistula 97 days after LT. The vascular injury resulted from the severe inflammatory reaction caused by the biloma involving the porta hepatis and was deemed not amenable to repair. The second graft loss was caused by hepatic artery thrombosis occurring on postoperative day 2. Despite the graft was showing good function, large necrotic areas were apparent at computed tomography scan, so a decision was made to relist the recipient for urgent retransplantation. Both patients were successfully retransplanted.

The rate of anastomotic biliary complications and ischemic cholangiopathy was comparable between groups (Table 2). In particular, only one case of IC was observed in the DCD group. This patient had a percutaneous biliary drain inserted before undergoing hepaticojejunostomy for a tight anastomotic stricture. Cholangiogram showed an isolated posterior duct stricture, likely representing an incidental finding. Patient was treated with a single balloon dilatation and has neither clinical nor radiological evidence of recurrence 8 months after the procedure.

**FIGURE 4:**
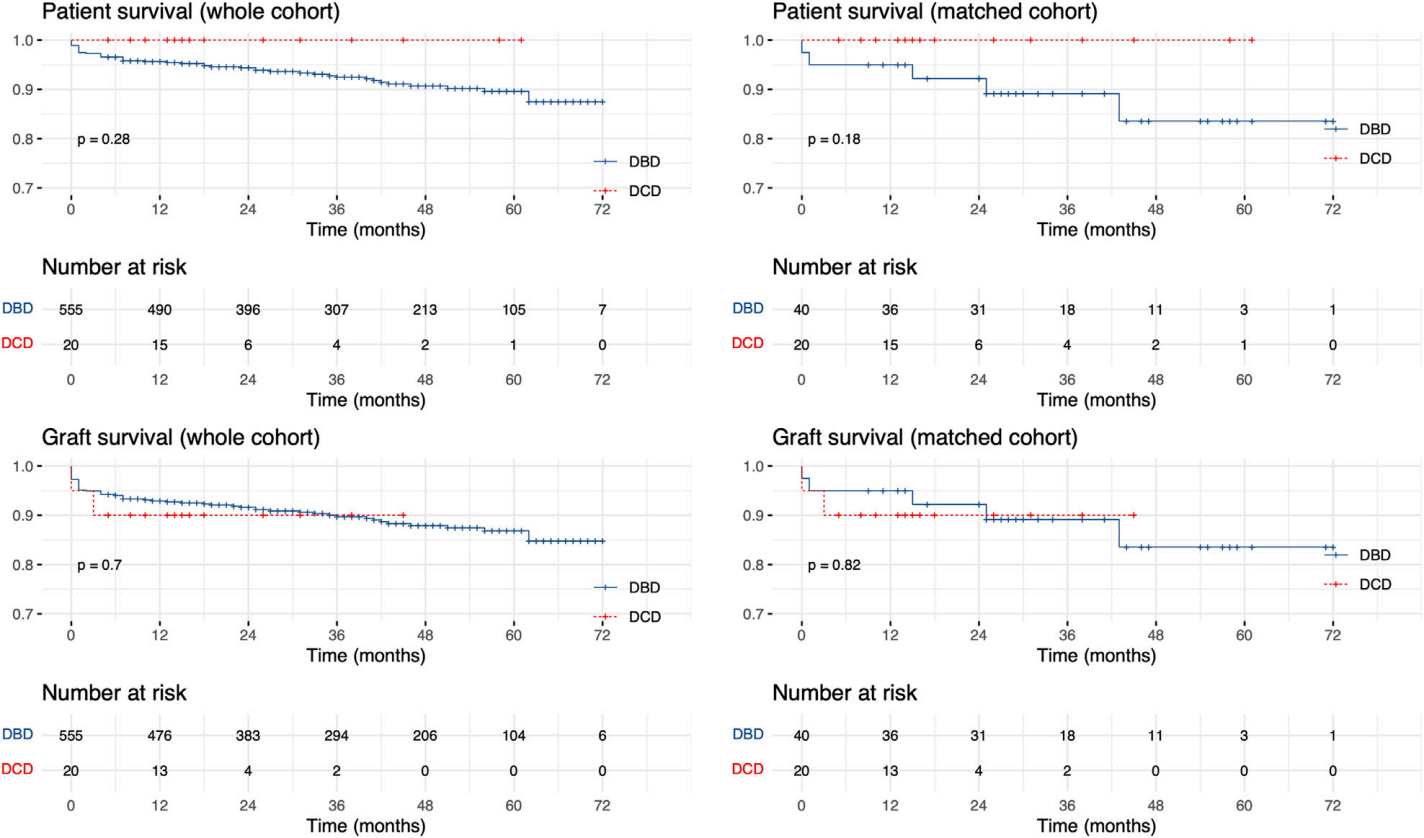
Kaplan-Meier patient and graft survival curves in the unmatched and matched cohorts.

Median follow-up was 40 (21, 56) and 15.5 (12, 27) months in the DBD and DCD group, respectively. Graft and patient survival was comparable between groups (Figure 4). In the matched cohort, 1-year patient survival in the DCD and DBD group was 100% (confidence interval [CI] = 100%, 100%) and 95% (CI = 88.5%, 100%), respectively, whereas 1-year graft survival was 90% (CI = 77.8%, 100%) and 95% (CI = 88.5%, 100%).

## Discussion

This study shows that a combination of A-NRP followed by D-HOPE is effective in preserving grafts from controlled DCD donors with prolonged WIT and allows obtaining comparable outcomes to DBD LT. These results appear to be even more remarkable if some peculiarities of the Italian setting are considered. Besides the 20-min no-touch time, which is unique among countries with active DCD programs (6), pre-mortem cannulation is not allowed in Italy, which further prolongs WIT due to the time necessary to cannulate femoral vessels and occlude the descending aorta (Figure 1). Furthermore, as the required 20 min of flat EKG recording are preceded by a variable time of pulseless electric activity, procured organs are exposed to a no-flow time that is frequently much longer than the 20-min no-touch time. If these livers were procured by ultra-rapid recovery and preserved by static cold storage, a poor outcome would be expected (1–4). In contrast, reconditioning and preservation by A-NRP + D-HOPE appears to allow obtaining good results, which are not different from those observed after DBD LT. It is worth noting that, despite initial concerns and logistic obstacles, our ∼60% utilization rate compares favourably with that observed in other realities (37, 38).

Overall, our results confirm the benefits of both A-NRP and D-HOPE in controlled DCD LT. As compared to ultra-rapid recovery followed by static cold storage, use of A-NRP has been associated with better graft function, lower rate of overall biliary complications and ischemic cholangiopathy, and improved graft survival (11–13, 15–17, 39). A recent large Spanish study has shown that use of A-NRP alone in DCD LT allows achieving comparable outcome to DBD LT (13). Additionally, use of A-NRP appears to positively impact on utilization rate and post-transplant function of other abdominal organs, especially kidneys (40, 41). On the other hand, DCD LT is the setting in which the advantages of end-ischemic D-HOPE have been more convincingly demonstrated (18, 19, 21, 42–44), with a recent randomized controlled trial showing that use of D-HOPE in this context is associated with a significant reduction of symptomatic non-anastomotic biliary stricture incidence from 18% to 6% (19). However, these data come from countries where local regulations allow usually limiting WIT to 10–15 min, which is much shorter than what is currently observed in Italy. Therefore, Italian centres have frequently considered to combine these two approaches. In Italy, successful use of controlled DCD donors by combining A-NRP and D-HOPE or normothermic machine perfusion has already been reported (9, 10, 22–24, 29), with a recent study by De Carlis et al.(25) showing that, despite longer WIT, outcome of liver grafts procured by this approach is comparable to those of DCD liver grafts procured by ultra-rapid recovery and SCS. To our knowledge, the present study is the first suggesting that the outcome of controlled DCD LT performed by combining A-NRP and D-HOPE, despite a functional WIT almost invariably exceeding 40 min, is not inferior to that of matched DBD LT.

Undoubtedly, these favourable results also issue from accurate donor selection and liver function assessment during A-NRP. In our experience, four (12.9%) initially accepted grafts were discarded based on parameters obtained during A-NRP. Different criteria for liver viability assessment during A-NRP have been proposed in different countries (8, 16, 17, 45, 46). Given the expected long WIT, we chose to adopt a modified version of the rather unrestrictive criteria proposed by De Carlis et al.(29). These criteria were not modified during study period and are still currently adopted at our centre. The good outcome observed in our series seems to confirm their validity. However, these data must be considered preliminary and future larger studies should investigate whether these criteria could be safely expanded further.

As LT outcomes are also influenced by recipient condition (26, 27), it is likely that recipient selection also played a role in achieving the good results observed in this series. This is the reason why, in order to allow a meaningful comparison, recipient characteristics were accounted for in the matching process. However, although initially DCD livers were preferentially allocated to low-MELD patients undergoing LT for HCC, the good results observed during the initial phases of this study fostered an increased confidence with DCD grafts utilization, which led to consider donor of progressively increasing age and to allocate DCD grafts also to patients with severe hepatic disease (Figure 3), without observing any detrimental effect on outcomes. This was also associated with an increasing number of DCD LTs per year (Figure 3). Overall, these findings are in keeping with the good outcome achieved and reflect how utilization of DCD liver grafts has become standard practice.

Limitations of our study include retrospective single-centre design and limited numerosity. Given the exploratory nature of this analysis, formal sample size calculation was not made. Also, as the majority of DCD LTs were performed in 2020–2021, follow-up was shorter in DCD group. Although 6-months minimal follow-up should have allowed capturing the majority of biliary complications, late-onset complications could have been missed. We are aware that an updated definition of functional WIT has been recently introduced (47). However, all cases included in this study were antecedent to its introduction and a retrospective recalculation of functional WIT was not possible. Finally, as all grafts included in this study were treated with D-HOPE, we could not evaluate the additional value of D-HOPE after A-NRP. It could be argued that use of machine perfusion could be omitted in selected cases, whereas additional viability assessment by normothermic machine perfusion could be indicated in others (48). In our experience, use of D-HOPE has been systematic for grafts meeting all viability criteria during A-NRP, which are those included in this series. So far, use of normothermic machine perfusion has been limited to cases characterized by doubtful evaluation during A-NRP (24), or in which logistics constraints imposed prolonging preservation time. Well designed and appropriately powered randomized studies are needed to define when and by which modality machine perfusion after A-NRP is indicated in DCD LT.

In conclusion, despite apparently prohibitive WIT, outcome of LT using livers from controlled DCD donors treated by a combination of A-NRP and D-HOPE is comparable to that of DBD LT, suggesting that a wider implementation of this approach could contribute improving the results of DCD LT and expand donor pool. Larger studies are required to confirm these findings, refine our evaluation process, and establish when and by which modality machine perfusion is indicated in this setting.

## Data Availability

The raw data supporting the conclusion of this article will be made available by the authors, without undue reservation.
